# A Practical Treatise on Abdominal Hernia

**Published:** 1846-07

**Authors:** 


### Art. XVII.

*A Practical Treatise on Abdominal Hernia.* By Thomas Pridgin Teale, F.L.S., Fellow of the Royal College of Surgeons, and Surgeon to the Leeds General Infirmary. With numerous Illustrations.—*London,* 1846. 8vo, pp. 383.

At the head of our table of contents stands the announcement that we are "critical and analytical reviewers." We need not, therefore, argue at any length that it is our especial function to analyse and criticise whatever is submitted to us for our perusal and candid opinion: and we are free to confess that when we have performed *both* of these duties, we lay down our pen with peculiar complacency and self-satisfaction. Yet it does occur to us at times to be foiled in this our laudable desire to fulfil our engagements with the public; which is of course no fault of ours, but is to be charged exclusively on those who favour us with their books. Some are pleased to write in such a mystical strain, and their meaning is wrapped up in such unintelligible language, or expanded into such vague generalities, as to defy our power of *analysis;* and we are forced to acknowledge ourselves blissfully ignorant of the great truths which are haply concealed from our ken. Others, again, are below *criticism;* and we are then sometimes tempted to lay aside our dignity, whilst we expend our spleen for the amusement of our readers and the wholesome chastisement of ignorant pretenders. But there is yet a third class of writers who no less baffle our efforts to criticise,—who, in short, write so cautiously and so well,—who advocate such sound practice, and broach such sensible theories, when they theorise at all, as to leave the *honest* critic without a handle for his weapon: for them his occupation is gone, and he is driven to the necessity of satisfying his readers by a simple analysis of the work he is reviewing,—itself no easy task, where neither tempting novelty is promised nor originality professed. To this last class belongs the book before us; which we at once pronounce to be unexceptionably good in every respect, whether literary or professional; and fulfilling to the utmost the stated object of its publication.

We are indebted for the present treatise, in its existing form, to the suspension of the work for which it was intended as a contribution.* It further appears from the author's statement, that the article on "Hernia," for this publication, was to have been undertaken by Sir Astley Cooper, whose decease, however, occurred before he had commenced the task. Mr. Teale was consequently requested to supply the article in question; but the work itself, in the meanwhile, became defunct, and our author was thus "kindly released from his engagement by the editor and publisher, when more than two years had elapsed since the last part of the work appeared." Additional matter and woodcuts, together with a reconsideration of the whole subject, "with a view to publication in an independent form," completed the preliminaries for the appearance of the volume before us.

As it is not very long since we devoted an article of some length to

* We presume The Cyclopædia of Surgery.

the subject of hernia, in reviewing the works of Lawrence, Malgaigne, Verdier, &c.,* we shall avoid as much as possible going over the same ground again; at the same time that we are anxious to do justice to a volume which, though unpretending in its extent and scope, possesses no ordinary merit as a concise systematic treatise on this important branch of surgery.

Mr. Teale's work is divided into two "Parts," the first of which comprises a "general consideration of abdominal hernia," and the second treats of "abdominal hernia considered in reference to its species and varieties." Each of these parts is subdivided into chapters, of which we now proceed to lay a brief analysis before our readers, together with such observations and illustrations as we may deem applicable.

Some remarks on the anatomy of the abdominal walls constitute an appropriate introduction to the surgery of these parts; and a clear understanding is at once established between the author and his readers as to the strict meaning to be attached to certain terms which admit of different interpretations,—a licence which should be inadmissible in the nomenclature of any branch of science. For the most part we approve of the definitions adopted by Mr. Teale; and especially of those which have reference more strictly to the ultimate anatomy of textures: such, for instance, as the substitution of "filamentous" for "cellular" tissue. The fascia iliaca and fascia transversalis are severally denominated "internal" and "external" abdominal aponeurosis; and the term "fascia" is restricted to "membranous expansions of areolar or filamentous tissue." This, again, we should not object to, if the sheet-like expansions of tendons were not thus deprived of their appropriate appellation: and on this account we should have preferred expressing the latter structures by the word "aponeurosis," and confining the term "fascia" to analogous and often continuous textures, such as that which invests the muscles of the leg, thigh, &c. The restriction of the title "subcutaneous filamentous tissue" to all superficial fasciæ, so called, has our entire concurrence.

After a brief notice of the "hernial apertures," our author proceeds to make some remarks on the "hernial sac," its mode of formation, forms, and varieties. With respect to the latter subject of inquiry, Mr. Teale adopts M. Cloquet's division into four primitive types; "the cylindroidal, spheroidal, pyriform, and conoidal;" and appropriate illustrations accompany this part of the letter-press. We need scarcely remind our readers that this classification, though very useful as far as it goes, by no means includes the infinity of forms which a hernial sac may present. The structural alterations which the sac undergoes in the course of time, is an important subject of consideration, as bearing upon the not infrequent seat and source of stricture; and the most important of these changes which Mr. Teale notices, is that of "the gradual conversion of the filamentous or areolar tissue into the white fibrous element." "I have," he says, "in my possession the sac of an old direct inguinal hernia, around the neck of which are numerous opaque, white, glistening fasciculi, precisely similar in their physical characters to those of which the dura mater is composed." We have ourselves dissected an old and large hernia presenting a similar

* Vol. XIV, p. 91.

character, though the glistening fibrous appearance was extended for some little distance on the sac, and consisted of scattered bands, which we were disposed to identify with the tendinous structures through which the hernia passed, rather than to the development of a new texture, or the modification of the texture of the sac itself.

The "Constituents of herniæ" (which is the subject of the next chapter) are almost as various as the contents of the abdomen: thus, not only have the membranous viscera contributed to such formations, but the uterus, kidneys, liver, &c., have been found as part of the contents of a hernial sac. One remarkable case Mr. Teale cites, in which Mr. Nourse removed the ovaries which formed a hernial tumour in each groin of a young woman in St. Bartholomew's Hospital. "The tumours had become so painful as to incapacitate her from pursuing her usual avocations, on which account she desired their removal. After the operation she enjoyed good health, but ceased to menstruate." Mr. Teale also points out the peculiar anatomical relations of the cæcum, as influencing its hernial state, remarking that "after it has descended as low as the internal ring, its posterior surface is the part usually first protruded into the inguinal canal. Hence, when the protrusion is of small size, it is constituted by a portion of the cæcum altogether devoid of peritoneum, and a true hernia without a sac is produced." Again, "as we can seldom be quite certain, before operating, whether the cæcum or the floating viscera constitute the hernia, we should bear in mind, even in small herniæ, the possibility of the cæcum being protruded, and also the anatomical peculiarities of this form of hernia; but still more should we remember the possibility of this being the case when the herniæ of the right side are unusually large." We may add that, as it is not at all unfrequent to find the cæcum entirely encased in peritoneum, and to a certain extent floating in the iliac fossa, the above relations of this portion of large intestine are not constant. There is considerable analogy between the ordinary relations of the cæcum and the bladder, as regards their serous investment; and the latter organ has been in many instances found to occupy the sac of a scrotal hernia. But this condition, as Mr. Teale remarks, can only exist under circumstances which have a tendency to produce an abnormal state of this viscus, and which enable it to find its way through the hernial apertures when undistended. Such are protracted repletion during pregnancy, or, more frequently, diseases of the prostate and urethra, which impair the contractile power of the bladder, especially in the aged; and it thus remains "in a flaccid state in the immediate vicinity of the abdominal or femoral rings." The absence of a peritoneal sac constitutes the analogy between this form of hernia and the cæcal protrusion. When once within the scrotum, the urinary bladder may become gradually distended, and retention of its contents may supervene. Our author reminds us that our earliest information respecting hernia of the bladder was derived from cases which had been mistaken, we presume for hydrocele, and punctured. The complication of calculus with cystocele has been met with in many instances; and the removal of the stone in some cases, and its spontaneous escape in others, has been known to effect a cure.

In a former article, already referred to, we entered somewhat at length into the statistics of hernia, and shall therefore pass by this division of our subject; especially as the authorities quoted are the same as those from which we drew our own materials. Great, we had almost said undue, importance is attached by our author to M. Malgaigne's calculations and tables.

The causes of hernia are divided by Mr. Teale into the "predisposing" and "exciting;" and of the former, inordinate size of the hernial apertures of course stands prominently in relief. We believe, with our author, that deficiency of the intercolumnar bands is a frequent cause of this abnormal size of the external ring; and we may add that the structure known as the "triangular ligament, or Colles's fascia," varies much in its density and development, and thus probably constitutes a negative predisposing cause to direct hernia. Amongst the ordinary *exciting* causes, the fair part of the community would do well to remember that all writers include "pressure from stays," by which the bowels are driven to take refuge where they may, so that the waist has its due proportion of compression.

The "effects of hernia" are briefly noticed, and then our author proceeds to the consideration of its various conditions. In speaking of reducible hernia, the circumstances which indicate the presence of omentum, as distinguished from intestine, are well set forth: its flabby and compressible character, its freedom from tension, uneven surface, ill-defined outline, and its restoration to its normal position in successive portions, render it highly probable that omentum is contained within the sac. Again, the importance of distinguishing between the symptoms resulting from inflammation, and those which indicate strangulation, are properly dwelt upon, and this more especially when the hernia is omental. "In inflamed hernia, the pain in the first instance is referred to the *body* of the tumour; while in strangulation, when the hernia is large, the *site of stricture* is more particularly the seat of pain. Again, in inflamed hernia, the ring is generally free from tension, whilst the swelling itself is tense." Mr. Key's high authority is then quoted to support the importance of this distinction, as, says that gentleman, "in the inflamed state of the omentum, without strangulation, the operation will afford no relief; on the contrary, it will aggravate the inflammation."

The obstruction of an irreducible hernia may, it is well known, produce symptoms very analogous to those of strangulation, although there be no stricture at the mouth of the sac. Attention has been recently called to this subject by Mr. Stephens,* but we have only room for our author's summing up of this gentleman's account, and the cautious advice which he appends thereto.

"The facts which have now been adduced are sufficient to prove, in the first place, that dangerous if not fatal, obstruction of the bowels may occur, in consequence of the intestine being fixed in an angular or distorted form by adhesions between the protruded viscus and the sac; and, secondly, that such a state of obstruction may sometimes be relieved by dividing these adhesions.

"But whilst these facts should never be lost sight of, nor be without their full influence in practice, it is necessary to use caution against committing the dangerous error of imputing to adhesions *all* obstructions occurring in hernial subjects not labouring under strangulation, and of rashly proceeding to attempt their removal by operation.

* Treatise on Obstructed and Inflamed Hernia.

"It must be remembered that the cases which justify this mode of proceeding are extremely rare; that the very adhesions which are supposed to have produced the obstruction have existed probably for years; and that it may depend upon some temporary superadded cause, as the presence of crude undigested food, which, by the natural efforts, or by medicine, may be removed. I may also add, that there is no necessity in these cases for hasty interference, since their progress is far from rapid; and consequently, that time is afforded for the judicious employment of less hazardous measures. Above all, it should be remembered that there is much greater danger in opening the sac of a hernia which is not strangulated, and thus exposing the general peritoneal cavity, than in opening the sac of a strangulated hernia, which is usually excluded from the general serous bag by plastic effusion near the mouth of the sac." (pp. 52-3.)

In the judiciousness of these observations every practical surgeon must coincide. In speaking of the periods of life at which strangulation may occur, Mr. Teale informs us that he had occasion to operate on a child under two years of age; and Dupuytren has operated as early as twenty days. These, however, are rare exceptions to a general rule; for it would appear from the statistical tables of Mr. T. W. King, quoted by our author from the Guy's Hospital Reports, that most herniæ exist for years before they become subject to dangerous strangulation. Our own observation certainly tends to bear out this conclusion, though we are disposed to refer the fact, in part at any rate, to the heedlessness occasioned, especially amongst the lower classes, by familiarity with a complaint to which they have long been subject. Such, at least, is our impression in looking back to the history of many cases which have come under our notice in hospital practice; yet we cannot deny that the more scientific explanation of Mr. King may have some share in producing the effect; viz. "a certain decline of vigour and health, connected with manifest deterioration of the great depurative organs of the body in persons of middle and advanced age, which renders the protruded part more liable to tumefaction, so that it becomes strangulated in consequence of its own ready turgescence."

The pathological effects of strangulation upon the involved textures are well described, and the deleterious nature of the effusion in diffuse peritonitis pointed out. It is true that this is a point acknowledged by all, and too well known to many who have experienced in their own persons, or watched in others, the virulent power of this poison. Yet the subject, as illustrated by Mr. Teale's own observation in one instance, is of such painful interest to a large proportion of our readers, that we venture, at the risk of being thought tedious by some, to extract it.

"One evening, at the dissection of the body of a patient upon whom I had operated for strangulated hernia, several surgeons were present. Of these, two attended one case of midwifery each during the following night, and a third three cases. The two patients attended by the first two surgeons died of puerperal fever. Two of those attended by the third surgeon also died; and his third patient escaped death from this formidable malady with the greatest difficulty, after having been in extreme danger several days. It is an important fact that no other cases occurred in the practice of these gentlemen." (p. 62.)

We could have wished Mr. Teale had mentioned how far these gentlemen meddled with the subject in question, or whether either of them abstained from handling the diseased parts. It is truly an impressive warning.

Constipation is justly regarded as so constant a symptom of the mechanical obstruction arising from strangulated bowel, that it is requisite the practitioner should be on his guard against being misled by the absence of this sign, when other evidence of a conclusive character is present. Thus, Mr. Tyrrell made the remark in several cases which came under his notice, that there was a free action of the bowels when perfect strangulation existed; and this he accounted for, no doubt correctly, by supposing that "the lower intestine had been much loaded at the time the hernia was strangulated; and that either the injection stimulated it to act, not once only, but three or four times, or that the sympathetic influence of the purgatives taken into the stomach had produced an action on the lower part of the bowels."* It is also, on the contrary, right to bear in mind, as our author remarks, that in strangulated omentum "constipation is less complete, for stools may, in most instances, be obtained by purgatives and clysters; yet sometimes constipation attends strangulated epiplocele, but it may, in most instances, be traced to peritonitis which has supervened."

In the chapter on the treatment of reducible hernia, the means proposed for effecting a radical cure are noticed, from Paulus Ægineta down to Velpeau; such as excision of the testicle; incision of the sac; excision, suture, and cauterization of the sac; ligature of the sac; acupuncturation, &c. No one of these operations has received the sanction of the profession, though some of them have been attended with partial success, and have thence acquired a transient reputation. Langenbeck,† for instance, "exposed the sac, and applied a ligature to the neck close to the ring, without detaching the sac further than was necessary for the application of the ligature." His statement of the result of this operation is that he had "performed it twelve times with the most successful results, and all the patients are capable of the heaviest labour without wearing a truss." The *modus operandi* of the ligature is analogous to that of a similar application on an artery. Yet the risk of this operation, to say nothing of the attendant suffering, caused it, in common with others, to be abandoned. The plans by injection (Velpeau's) and acupuncture (Bonnet's) are briefly dismissed by our author, with the remark that a repetition of them would be altogether unjustifiable; their results indeed can lead to no other conclusion. M. Belinas's mode of treatment by the introduction of gold-beater's skin into the mouth of the sac seemed to promise a more favorable prospect of success; but although the required adhesive inflammation appeared to be established without any important constitutional disturbance, it seemed very questionable in many of the cases whether a radical cure was effected; in some it was clear that the neck of the sac remained patent, as the herniæ reappeared. However, as Mr. Teale justly remarks, "the obliteration of the sac affords but a feeble barrier to a fresh hernial descent, and only a very slight advance is thereby made towards a radical cure of the disease." In favour of the practice of applying a ligature on the sac and its envelopes, the success of Desault and Thierry may be quoted; but it has been condemned by Sir A. Cooper, and Scarpa's opinion was not favorable to it. Mr. Teale regards it as a means whereby a radical cure *may* be obtained, but considers the attendant risk and suf-

* St. Thomas's Hospital Reports, as quoted by Mr. Teale.
† Bibliothek für die Chirurgie, Band ii, quoted by Mr. Teale.

fering as a sufficient bar to its introduction into general practice. Lastly, this plastic era has induced MM. Velpeau and Gerdy to propose operations based upon this principle for the cure of reducible herniæ. Our space will not allow us to analyse this part of the subject, for the details of which we refer to our author, in whose summing up we concur, viz. that

"The operation can only be considered justifiable when well-directed attempts at palliative treatment have failed, and the inconvenience and suffering occasioned by the hernia are so great as to interfere seriously with the patient's avocations, comfort, or health." (p. 88.)

The treatment of 'irreducible hernia' constitutes the subject of a short chapter, in which our author wisely recommends that the attempt to render this form of the disease reducible should be limited to cases of comparatively recent date and moderate size, and which are irreducible from hypertrophy; in such he advocates, where the system will bear it, the free and repeated abstraction of blood, the exhibition of purgatives, the employment of cold and compression, and the recumbent posture, with a rigid diet. All these remedial agents have been applied more or less in combination with marked success, where no permanent mechanical obstacle to the reduction existed; but when such is the case, the support afforded by a truss with a hollow pad is unquestionably the appropriate treatment to adopt.

Amongst the "agenda," when a case of strangulated hernia presents itself, the *taxis* stands first, and Mr. Teale very properly devotes some space to the necessary cautions to be observed in the employment of this powerful remedy. Let our younger readers take warning by the following illustration which he gives, of the injuries which may be inflicted by the injudicious use of the taxis.

"A man, aged 39, was admitted into the Glasgow Infirmary, having been twenty years the subject of a reducible hernia, which had been strangulated ten hours. During the greater part of this time a surgeon had made powerful and continual efforts to return the displaced parts, and on his admission into the hospital, the taxis was again rather forcibly employed. When the patient was seen by Dr. Macfarlane, he considered it improper to make any further attempts at reduction. The scrotum was much swollen and discoloured. When the sac was opened, not less than a pound and a half of dark-coloured blood escaped, a considerable quantity of which was pressed from the depending part of the scrotum. The hernia consisted of a large portion of omentum, which was covered with coagulated blood, and of nearly two feet of intestine. The omentum was bruised and lacerated, and the protruded gut was almost wholly separated from the mesentery. It contained several rents, which passed in a longitudinal direction; and into each of these openings two or three fingers could be introduced." (p. 96.)

But not only is force to be deprecated in the employment of the taxis, but its protracted use, even with due regard to care in other respects, is often fatal to the patient, by the loss of time which it entails. So strongly, indeed, are we impressed from experience with the importance of an early recourse to the knife, that we do not hesitate to assert our belief that a large number of the cases which prove fatal after operation owe this result to the prolonged employment of the taxis, and consequent waste of precious time. We are persuaded, that more success would attend the operation for strangulated hernia, if attention were paid to this circumstance; and that the unfavorable issue of cases subjected to the knife is more generally due to the condition induced by protracted strangulation, than by exposure of the contents of the hernial sac. We would even go farther, and prefer to err in occasionally operating where the stricture might perchance have yielded to persevering taxis, than risk the sacrifice of our patient by dangerous delay. Mr. Teale is scarcely less decided in his opinion on this subject; for he remarks that "of all the causes which determine the fatal issue of strangulated hernia, delay is the most frequent and dangerous." In speaking of position during the manipulation of taxis, our author says that he has on several occasions succeeded in reducing herniæ by adopting Lisfranc's suggestion of making the walls of the abdomen tense, when he has failed whilst they were relaxed. It certainly is true that the capacity of the apertures, from their peculiar conformation, admits of much less alteration by position than is generally supposed; and Lisfranc grounds his recommendation on the assumption that "it is much easier to push a substance through an opening of a given size in a tense than in a relaxed membrane:" this it is difficult to gainsay, and it is well to try the second method when the first fails.

We must pass over the clear and excellent directions given for the employment of the taxis and its adjuvants, recommending them to the careful perusal and study of our readers, and proceed to the consideration of the operation itself; and the first question which presents itself is, whether the sac should be opened, or an attempt be made to relieve the stricture without this ulterior step. Our author sets out with assuming that the fatality of cases, in which the ordinary operation is performed, is in part due to the exposure and manipulation of the protruded viscera, and ably advocates the alternative of dividing the stricture external to the sac. It is true that if this attempt fail, the sac may then be opened, and therefore, if care be taken not to push up the still strictured hernia with its sac into the abdomen, there can be no objection to pursuing this course. We repeat our conviction, however, that much of the mischief which has been attributed to the exposure of strangulated intestine, is really due to the impression made by the stricture, and thence propagated even to the interior of the abdomen. On this still mooted subject we allow Mr. Teale to speak for himself.

"My experience certainly justifies me in recommending this mode of operating whenever it is practicable, provided the necessary precautions are taken against incurring the evils to which the operation, when carelessly performed, might be exposed; and, in order to avoid these dangers, it is important to be able in the first place to recognize the symptoms which indicate the occurrence of gangrene, or of a state verging towards it; and, secondly, to guard against employing an improper degree of force for the purpose of replacing the hernia after the stricture has been divided."

We need scarcely add, that in this operation the object is, after division of the stricture, to empty the sac of its contents *without* returning the former; where this is effected, the surgeon may be pretty well satisfied that he has accomplished what he desired. Again, as the signs of gangrene, either local or general, are usually of a satisfactory character, and, as we have already remarked, the alternative of opening the sac always remains where any doubt exists, we are well disposed to concur in the opinion of our author, that the operation in question should be first attempted in most large herniæ, and in many of middle size; though it is more rarely admissible in small herniæ. At the same time, the cautious advice of Mr. Key cannot be too strongly enforced, viz. that, after division of a stricture external to the sac, no more pressure should be used than would suffice for reducing a hernia where no strangulation exists. We learn from Mr. Teale that it is likewise Mr. Liston's practice to try, in all recently strangulated herniæ, to divide the stricture without opening the sac. Sir A. Cooper's modification of this operation in cases of large herniæ, should be borne in mind; it consisted in making a small opening into the sac, and dividing the stricture on a director from within; though it is of course preferable, where practicable, to avoid even this interference with a large hernial sac.

The tabular arrangement supplied by Mr. Teale of 32 cases, in which the stricture was divided external to the sac, certainly presents us with an unusual average of success, as 27 recovered; of these, 18 were femoral herniæ, 11 inguinal, and 3 umbilical.

The directions given by our author for conducting the operation for strangulated hernia are judicious, but do not present anything which calls for remark. He speaks highly of a winged director, devised by Mr. Turner of Manchester, "which resembles an ordinary director attached to the upper surface of a thin, slightly convex plate of steel, terminating in a rounded extremity like the finger-nail, and allowing the grooved director to project about an inch beyond it." The object of this contrivance is to protect the viscera from injury.

The treatment of the intestine under the various conditions it may present, next comes under consideration; and Mr. Teale lays down as a rule, that "no degree of discoloration of the intestine, short of its vitality being extinct, forbids the replacement of the part within the abdomen." This is, indeed, the recognized rule of practice, though the determination of the vitality or non-vitality of the affected part is not so easily arrived at in all instances. The directions given by our author for the solution of this problem are the best which can be followed. He approves of Sir A. Cooper's recommendation, to close a single, small perforation of the intestine by a fine silk ligature; and, after cutting off both its ends close to the knot, to replace the intestine within the abdomen. With respect to the delicate question of what is to be done with adherent intestine, Mr. Teale recommends that, when recent, the adhesions should be destroyed by the finger, or handle of the scalpel, provided the intestine be not in a gangrenous state, and that "old organized adhesions," when of moderate extent, should be cautiously divided by the knife; if even a portion of the sac be returned with the intestine no mischief is likely to ensue therefrom. When a large portion of omentum presents itself, Mr. Teale advocates the practice of cutting it away, though perfectly healthy, as he considers there is less risk in this operation, than is to be encountered by replacing it after exposure and strangulation. In accomplishing this removal, he further advises the employment of a temporary ligature, to prevent retraction of the remnant, and facilitate the subsequent securing of the vessels. Purgatives should be avoided for some hours afterwards.

An excellent chapter on "reduction in mass" succeeds. The conditions pointed out as the "chief, if not the essential, ones which favour the accident," are "a contracted and indurated state of the neck of the sac, and a wide hernial aperture." Of course, in these cases the neck of the sac itself must be the seat of stricture, and their history does not seem to indicate that great violence in the employment of the taxis is necessary to this result; indeed, it would appear that it may even occur spontaneously, as in an instance which came under Dupuytren's notice. The sudden return of the tumour, as Mr. Teale remarks, in place of its gradual diminution under pressure, attended by the characteristic gurgling sound, together with the persistence of symptoms of strangulation, constitute grounds for suspecting the occurrence of reduction in mass; and this receives further confirmation by the external ring being found large, and unoccupied. The presence of a tumour in the abdomen, in the vicinity of the inguinal canal, is a further corroborative but not a necessary symptom; it was existing in one of Mr. Luke's cases, when the patient himself had returned the hernia. The treatment adopted and recommended by the last-named surgeon is approved of by our author, viz. by exploratory examination where the accident is suspected, and the completion of the operation in the ordinary way, by drawing down the sac, and enlarging the ring if necessary. A useful tabular view of this class of cases follows; for the details of Mr. Luke's cases, we refer our readers to his excellent paper on the subject in vol. xxvi of the 'Medico-Chirurgical Transactions.'

The concluding chapter of the First Part of the work is on "Intestinal Fistula, consequent upon gangrenous or wounded Hernia." The opinions of Scarpa on this subject are quoted, and an interesting case is referred to which is narrated by that eminent surgeon, illustrative of the mode of communication after separation of a coil of intestine; the medium of communication, in this instance, was a membranous funnel formed by the hernial sac. A just compliment also is paid to Mr. Travers's original and excellent treatise, which, though an acknowledged and high authority on the subject of 'Injuries of the Intestines,' has not received that general attention and study which its merit deserves. The anatomical characters, effects, complications, and treatment of intestinal fistula follow; in the details of which our space will not allow us to follow our author. He points out that the principal objects to be attained by surgical interference are, the removal of the obstruction caused by the projecting ridge or valve developed at the point where the two orifices of intestine are *parallel* to each other, (such being their usual if not invariable relation, after the sloughing of an intermediate portion), and to close the external wound, when the proper time shall have arrived. To carry into effect these and other essential parts of the treatment of the above class of cases, clear and judicious directions are given, including an ample view of Dupuytren's operations with the seton and enterotome.

We now come to Part II of the work, which, as we have already stated, treats of abdominal hernia, in reference to its species and varieties. The opening chapter is on "Inguinal Hernia," the anatomy of which is accurately given and nicely illustrated. Mr. Teale appears to coincide with Mr. Curling in the view that gentleman takes of the structure of the gubernaculum testis; stating that to him "attaches the merit of having proved by the microscope that a great portion of the gubernaculum consists of muscular fibres; that its attachments are identical with those of the cremaster; and that through the agency of this structure, which must be regarded as the fœtal cremaster, the descent of the human testicle is accomplished." In the statistics of inguinal hernia, and the causes to which it is referrible, the tables and calculations of Cloquet and Malgaigne are laid under contribution; but as these have been discussed in our previous article, already referred to, we shall not stop to devote more time to them in our present. When speaking, in the succeeding chapter, of oblique inguinal hernia, Mr. Teale gives a preference to the French term, "interstitial," as applicable to the *incomplete* herniæ, or those which have not projected beyond the external ring. In this form of rupture, in its early stage, the importance is indicated of investigating with the utmost care the site of the internal ring, where no external swelling exists, but when the "symptoms of intestinal obstruction are present without the cause of the obstruction being apparent." We quite believe that in these obscure cases, as our author remarks, a fatal termination not infrequently ensues, without the real cause of the symptoms being suspected.

In discussing the comparative frequency of oblique inguinal hernia in women, M. Malgaigne's novel views and inferences are noticed, but without comment. The characteristics by which this form of rupture is distinguished from direct and femoral hernia, as well as from hydrocele of the spermatic cord, are pointed out: we perceive that our author agrees with all experienced and practical surgeons in adopting, in all cases of inguinal hernia, the safe recommendation of Sir A. Cooper, to divide the stricture directly upwards. The complication of inguinal rupture with hydrocele of the cord in many recorded cases renders it desirable, where symptoms of strangulation exist, to cut down carefully upon the tumour: a case is quoted by Mr. Teale, in which Mr. Liston discovered a small hernial sac by the side of the hydrocele; a similar case is recorded by Scarpa; and others have occurred in the practice of many other surgeons. Mr. Lawrence's diagnostic test is, "fluctuation of the watery tumour at its lower part; its imperfect removal under pressure, so that the cord can never be felt in a natural state, and sometimes a visible enlargement of the inguinal canal and its neighbourhood, when the fluid is pushed upwards." If there be a question between the existence of irreducible omental hernia and hydrocele of the cord contained in many cysts (the diffuse hydrocele of Pott), our author recommends the employment of acupuncture, by which "the presence of fluid may be surely and safely tested."

Another source of error may exist in the presence of air in the intestine, which, from its translucency, may deceive the incautious practitioner into the belief that he has to deal with a hydrocele. An interesting case, which occurred in the practice of Arnaud, is cited by Mr. Teale, but we prefer illustrating this delicate point of diagnosis by quoting one of his own.

"A boy was brought to me at the Leeds Infirmary with a tumour of the scrotum of large size, tense, and elastic; the integuments being very thin from the distension which it occasioned. On examination by the aid of a candle, in the presence of several pupils, it was found to be as translucent as any hydrocele which I had seen. Whilst examining it, I observed that there were two opaque lines intersecting the tumour diagonally; which, by pressure, were made to a certain extent to alter their situation. This circumstance, together with the comparative lightness of the tumour, and slightly tympanitic condition which I detected, excited my suspicion as to the disease being a hydrocele, and justified me in treating it as a hernia. The use of the taxis showed that the diagnosis was correct, and that the tumour was a hernia greatly distended with flatus; for by pressure the distended bowel was with some difficulty replaced within the abdomen. The opaque oblique lines indicated most probably the point of contact of distinct folds of intestine." (p. 253.)

Our space will only allow us to enumerate the heads of the other diseases which more or less simulate hernia, but which are to be distinguished by the rules which our author lays down: they are, "hydrocele communicating with the abdomen, varicocele, chronic abscess descending through the inguinal canal, adipose tumours, inflamed lymphatic gland, a testicle retained within the inguinal canal, and hæmatocele of the tunica vaginalis."

The *treatment* of oblique inguinal hernia forms the subject of the succeeding chapter, in which we have some good suggestions afforded on the subject of trusses. In old ruptures, where the posterior wall of the canal has been encroached upon, in consequence of the long standing and weight of the protrusion, by which the rings are rendered parallel, Mr. Teale approves of a triangular pad, "the inferior angle being most acute, and corresponding to the upper surface of the pubes, whilst the inferior border of the pad accurately corresponds with the fold of the groin." This pad, he adds, "should be well stuffed, having its greatest convexity along its superior border; so that, whilst its inferior angle rests gently upon the pubes, the superior border may make a more decided pressure upon the muscular wall." To the form of the spring our author attaches great importance, both as regards the efficiency of the truss, and its comfort to the wearer. He considers, and we agree with him, that it should adapt itself accurately to the oblique bearing of the back of the pelvis and lower part of the abdomen, as well as to the "vertical outline of the haunches." To attain this object, Mr. Teale employs springs with two reversed curves; the posterior of which has its convexity directed downwards, and the other, at the anterior extremity, is curved in the reverse direction. This arrangement is at once rendered intelligible in the text by the never-failing accompaniment of a woodcut. The *double* common truss is constructed on the same principle, presenting a triple curve. This chapter closes with a brief account of the operation for the interstitial and scrotal forms of oblique inguinal hernia, with and without opening the sac. We have already, in an earlier part of this article, referred to our author's views on the relative merits of these operations.

In the chapter which is appropriated to "hernia of the tunica vaginalis," the varieties of this form of the disease are brought before us, viz. that in which the protrusion occupies the sac of the tunic, in front of the testicle; that in which the hernia has the same serous relations, but to the exclusion of the testicle, which organ may either remain within the abdomen or have descended to the inguinal canal; and the *funicular* variety of the same, in which the vaginal tunic still forms the hernial sac, but is cut off from the testicle by intervening adhesion. This, as well as the other forms, may usually, but not invariably, be traced to an early period of life; though the ordinary term "congenital," is inapplicable, inasmuch as the disease does not occur till after birth. It is the last-mentioned variety which was denominated by Sir A. Cooper "encysted hernia of the tunica vaginalis;" its relation to the testicle is not invariable; and, where lying in front of that organ, might easily be mistaken for oblique hernia in its more ordinary form. In the treatment of these cases during infancy Mr. Teale prefers the employment of a pad of ivory invested with an appropriate and easily-shifted envelope, which should be frequently changed for the sake of cleanliness. But when a hernial protrusion exists in conjunction with retention of the testicle within the inguinal canal, "a truss with a hollow pad must be employed." The existence of stricture at the mouth of the sac itself, when this form of hernia is strangulated, usually precludes, as our author admits, the practicability of operating without exposure of its interior.

The nature and treatment of "direct inguinal hernia" occupies the next chapter. Though presenting nothing original, to justify a lengthened quotation, we consider the directions given by our author for conducting the operation in this form of rupture when strangulated so judicious, and as constituting so fair an illustration of his practice and style, that we transcribe the passage which contains them.

"As the surgeon is unable to pronounce with certainty that the hernia is direct, it is his duty, whenever he operates for a strangulated inguinal hernia supposed to be direct, to proceed under the constant apprehension that the hernia may possibly be oblique. If the local and general symptoms are such as to warrant his attempting to relieve the stricture without opening the sac, a small incision should be made over the upper part of the tumour, from above downwards, so as to expose the external ring and the upper part of the fascia of the cord; an opening of small extent being next made into the latter, a flat director (Key's) should be passed upwards under the external ring, and, if it exert any material pressure on the tumour, a few of its fibres should be divided by the bistoury. If, however, it be found that the stricture is not formed by the external ring, and if the cremaster be found spread over the tumour, it should be turned aside by the point of the director, or, if necessary, divided by the knife, in order to ascertain if the next envelope present the smooth, firm, resisting character of an aponeurotic membrane; and, should this be the case, the membranous covering should be cautiously opened, and a flat director insinuated beneath it towards the abdomen, when any of its fibres which may appear to exert a constricting influence may be divided by the blade of the knife directed upwards. If, on the contrary, after opening the fascia of the cord, and turning aside the fibres of the cremaster, a loose filamentous tissue, more or less loaded with fat, present itself, the operator may presume that this is the subserous tissue pushed before the sac through a rent in the aponeurotic structures. It then becomes necessary to search for the upper edge of this aperture, and, after insinuating the director beneath it, to divide it upwards, and thereby remove the stricture. If, however, it is now found that these parts, external to the sac, have not been the seat of stricture, the operation must be completed by enlarging, if necessary, the external incision, and dividing the subserous tissue and the sac.

"Lastly, the director must be introduced within the stricture from the interior of the sac, and the constricting band divided; the operator always bearing in mind that he must direct his incision in this, as in all other forms of inguinal hernia, *upwards* from the middle of the mouth of the sac; and no presumptuous deviation from this rule, from a confident feeling in his own powers of diagnosis, can be justified under any circumstances." (p. 295-6.)

As in the preceding chapters, that which follows on "femoral hernia" is introduced with some notice of the anatomy of the region and parts concerned. In his description of the femoral sheath, and the walls of the femoral ring, we cannot help recognizing what we consider factitious anatomy rather than the simple reality. In the first place we should question, were we so disposed, the continuity of the femoral sheath and the fascia transversalis; but this may pass, for if we entered on this subject at all, it would be requisite clearly to define what is meant by the sheath of a vessel, which we hold to be anything but identical with a tendinous or fascial bed in which it may chance to lie. But, as regards the crural ring itself, the anatomy, as far as we have ever been able to make it out, is very simple; this space being bounded by the iliac fascia, Poupart's ligament, the vein, and Gimbernat's ligament; and closed by the cribriform fascia, extending from the last-named boundary to the femoral vein. As to the existence of any septum, properly so called, between the ring and vein in the normal condition, we know of none, nor can we conceive what purpose it could answer. It is true that the cribriform fascia (of Sir A. Cooper) may be and is so prolonged by continued and gradual pressure, as to form an investment of femoral hernia; and it is thus that the sac is separated from the vein. The condensed structure which occupies the saphenic opening is altogether independent of the femoral sheath, by which we mean the close cellular investment of the vessels, as well as distinct from the fascia transversalis, which can hardly be said, under any circumstances, to extend beyond Poupart's ligament. However, we are aware that we are on debatable ground; and our only desire is to enter our protest against the tendency which certainly exists with some writers of complicating practical anatomy by describing things as they think they ought to be, rather than as they really are. But this is not a charge we can bring against our author; although we think his anatomy of the femoral ring and sheath might a little puzzle a beginner. Again, we may remark, that though it is doubtless true, that the adhesion of the reflected border of the saphenic opening to the cellular sheath of the vessels is a barrier to the descent of a hernia *within* the fascial envelope, still we do not think that sufficient importance is attached to the ingress and egress of the blood-vessels and lymphatics at this part, by which the superficial and deep parts are, as it were, closely pinned together, and the protrusion is thus forced to take an upward direction, pushing before it the cribriform and saphenic fasciæ. A remark of our author's which follows we quite concur in, viz. that the tumour in crural hernia "usually projects over the semilunar edge of the femoral aponeurosis below, and over Poupart's ligament above, encroaching in the latter direction on the site of inguinal hernia. But," he adds, "this encroachment is not produced by the entire tumour turning upwards, as has been frequently represented, but is simply the result of the general enlargement of the body of the sac in comparison with the aperture through which it has escaped." Mr. Teale informs us that he has operated on a femoral rupture in a male, which was as large as two fists; this size, however, is very rare; as are other instances in which the relation of the hernial sac to the femoral vessels is described as different from that which we have indicated.

The complications of femoral hernia noticed by our author, and which it is necessary for the surgeon to be acquainted with and prepared for, are the coexistence of inguinal rupture, the presence of serous cysts, and enlarged lymphatic glands. He mentions an instance which recently came under his notice, of the rare complication of two inguinal and two femoral herniæ in the same subject.

The seat of stricture in femoral hernial has been the subject of considerable dispute and difference of opinion; and we do not doubt that the disputants have been all more or less in the right, though we are free to confess that our own observation leads us to place it in by far the largest proportion of cases in the sharp border of Poupart's ligament which overhangs the ring. Mr. Teale ascribes it to the femoral sheath in most instances, and quotes Sir A. Cooper and Mr. Key as confirming this view. It is certainly a very delicate point to decide, inasmuch as the adhesion of the so-called sheath to the posterior aspect of Poupart's ligament, (the "intermixture of aponeurotic fibres," to use our author's own expression), entirely destroys the individuality of these structures at this point; and though it may be true that these aponeurotic fibres below Poupart's ligament *aid* in strangulating a protruded portion of gut, yet we very much question whether their division alone would suffice to relieve the stricture. In short, we do not recollect to have witnessed a case in which the stricture was relieved (when its seat was the fibrous ring in front, and to the inner side of the neck of the sac,) without a more or less decided division of the posterior border of Poupart's ligament.

When reviewing M. Malgaigne's work we noticed his test for diagnosing crural as distinguished from inguinal hernia, we need not therefore now repeat it; the directions are given at length in the volume now before us. Nor is it necessary to notice the distinguishing characteristics of varicose veins and psoas abscess, which can scarcely be mistaken for hernia where ordinary caution is employed in conducting the examination of the patient. The "statistics and causes" of femoral hernia we may likewise pass by; but on the treatment of this form of rupture we have a few words to say before we leave it.

The employment of trusses in crural hernia is a palliative measure, which conduces very importantly to the patient's comfort and safety; but they effect little else, on account of the nature of the bounding textures of the ring, which tends to render its abnormal size permanent. As Mr. Teale remarks, it is important that the pad for these trusses should not be of large size, as it is then so readily displaced by the action of the adductor and flexor muscles of the thigh; and it is also important that it should not rest on the pubes. The following are his directions for the construction of a common single truss for reducible crural hernia.

"It should have the pad of a triangular form, narrow transversely, and somewhat elongated from above downwards; its base corresponding with the edge of Poupart's ligament. The pad should be of size sufficient to close the saphenous opening, but should only extend to a very limited distance beyond the borders of this aperture. Its convexity should constitute a rather prominent ridge, directed from above downwards, situated a little towards the pubic side of the pad, commencing about a finger's breadth below its upper edge, and extending downwards to the apex, towards which part it should gradually diminish. A pad thus constructed gently closes the external aperture, namely, the saphenous opening; and its most intense pressure is directed to the pubic side of the vein." (p. 327.)

Surgeons should not think themselves, as many do, above this purely mechanical part of their art; for how could a mere mechanist construct an apparatus, which it requires an accurate knowledge of anatomy to render available? Where a surgeon can add to his professional acquirements a practical familiarity with mechanics, he possesses a considerable advantage over another who is without this knowledge, not only in the use of his hands, but in the construction of his appliances to boot.

In the treatment of irreducible femoral hernia, our author strongly recommends a hollow pad, consisting of a "flat ring of metal supporting a concave metallic plate inclosed in a bag or cap of wash-leather. The metallic ring should be adapted to the size and form of the particular hernia, so that it may rest upon the parts immediately surrounding the tumour, without exerting any direct pressure upon it; whilst the leathern cap, spread over the opening of the ring, should possess such a degree of concavity as to enable it to support and to exert a gentle pressure upon the hernia." This form of truss Mr. Teale has not found obnoxious to the objections which Sir A. Cooper had to their employment in this form of irreducible hernia.

The operative course to be pursued when strangulation exists is judicious; and we are willing to acquiesce in our author's recommendation, that the stricture should be divided, where it can be done satisfactorily and safely, without opening the sac; and we quite believe that the surest rule for the division of the stricture is to direct the edge of the bistoury upwards and forwards.

In the chapter on "umbilical hernia," there is not much to attract our attention. A truss invented by Mr. Eagland, of Leeds, and which was highly commended by Mr. Hey, is that which our author prefers in ordinary cases of umbilical rupture. It is figured in the work before us, and consists of a convex cork pad, and a horseshoe spring attached by a hinge to either side. When there is a very lax and pendulous state of abdomen, Mr. Teale advises that the pad should be attached to "a broad plate of steel of very moderate concavity;" or if necessary, an ivory compress retained by adhesive plaster may be employed. The frequent fatality of this form of hernia, when strangulated and subjected to operation, certainly justifies the remark that the sac should not be opened, if the stricture can possibly be relieved without this step.

The remaining chapters of the work treat successively of "ventral, obturator, ischiatic, perineal, vaginal, pudendal, and diaphragmatic herniæ." Mr. Teale is enabled to add but little from his own personal experience to our information regarding these less common forms of hernia. He narrates one fatal case, which occurred in his own practice, of ventral hernia. In protrusions at the anterior wall of the vagina, a truss, "consisting of a compress supported by a spiral spring, so as to press upon the external labia," is spoken of in the highest terms, as applicable where an internal pessary cannot be borne; this instrument is also manufactured by Mr. Eagland, of Leeds.

We must here bring our analysis to a close, hoping that our readers have seen sufficient to satisfy them that our commendation has not been misplaced, but fearing likewise the fulfilment of our own hinted prediction, that the task we are concluding is one which, from its monotony, it is not most easy to render agreeable to the reader. Yet, although we cannot award to Mr. Teale the merit of originality, to which indeed he does not profess to lay any claim, we consider that he is entitled to our warmest thanks for having given to the profession so valuable a text-book in this important and intricate branch of surgery.

The subject is handled with that practical familiarity which cannot fail to engender confidence, and at once to instruct and interest the reader. The style is simple, terse, and pure, without bordering on dogmatism on the one hand, or falling away into vagueness or indecision on the other. Altogether, we may repeat, that we have rarely had it in our power to recommend a work with such unmixed confidence and satisfaction to our readers: and this we do most cordially to all classes, but especially to students and junior practitioners, who will find it an invaluable and unfailing guide in their early operations and maiden difficulties.

---

